# Sequencing Degraded RNA Addressed by 3' Tag Counting

**DOI:** 10.1371/journal.pone.0091851

**Published:** 2014-03-14

**Authors:** Benjamín Sigurgeirsson, Olof Emanuelsson, Joakim Lundeberg

**Affiliations:** Science for Life Laboratory, School of Biotechnology, Royal Institute of Technology (KTH), Stockholm, Solna, Sweden; University of Connecticut Health Center, United States of America

## Abstract

RNA sequencing has become widely used in gene expression profiling experiments. Prior to any RNA sequencing experiment the quality of the RNA must be measured to assess whether or not it can be used for further downstream analysis. The RNA integrity number (RIN) is a scale used to measure the quality of RNA that runs from 1 (completely degraded) to 10 (intact). Ideally, samples with high RIN (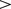
8) are used in RNA sequencing experiments. RNA, however, is a fragile molecule which is susceptible to degradation and obtaining high quality RNA is often hard, or even impossible when extracting RNA from certain clinical tissues. Thus, occasionally, working with low quality RNA is the only option the researcher has. Here we investigate the effects of RIN on RNA sequencing and suggest a computational method to handle data from samples with low quality RNA which also enables reanalysis of published datasets. Using RNA from a human cell line we generated and sequenced samples with varying RINs and illustrate what effect the RIN has on the basic procedure of RNA sequencing; both quality aspects and differential expression. We show that the RIN has systematic effects on gene coverage, false positives in differential expression and the quantification of duplicate reads. We introduce 3' tag counting (3TC) as a computational approach to reliably estimate differential expression for samples with low RIN. We show that using the 3TC method in differential expression analysis significantly reduces false positives when comparing samples with different RIN, while retaining reasonable sensitivity.

## Introduction

RNA extraction is one of the first steps in gene expression profiling experiments whether the assay is qPCR, microarrays or RNA sequencing, but RNA molecules, however, are relatively unstable and susceptible to degradation. Without specialized protocols [1], having good quality RNA samples is considered paramount for meaningful experimental results. Thus it is imperative to assess the quality of the RNA prior to any downstream analysis. The RNA integrity number (RIN) algorithm has become a widely accepted standard for quality measurement of RNA and was shown to be more meaningful and more robust than prior methods of UV spectroscopy and pure ribosomal RNA ratios [2], [3]. The RIN is developed from a learning algorithm that uses the whole trace from capillary electrophoresis (Bioanalyzer) rather than just the ratio of the large ribosomal peaks, though the RIN is still heavily dependent on that ratio [2].

For RNA samples to be considered of high integrity and to pass quality control the RIN usually must be higher than 7 or 8. Despite following standard RNA sample handling procedures, acquiring samples of high quality can prove difficult. This is especially true for post mortem, forensic and certain clinical samples. We thus find it of utmost importance to determine how samples with different RINs compare in RNA sequencing, what constitutes a RIN quality threshold for sequencing and what measures can be taken when handling samples with low RIN both in sample preparation and in data analysis.

For qPCR, samples with low but similar RIN have comparable expression profiles but low RIN samples have significantly lower expression compared to high RIN samples [3]. For microarrays, short genes and 5' end probes show highest effect on gene expression due to degradation [4]. Originally, when estimating transcript abundance in RNA sequencing, it was assumed that the reads were evenly distributed along the transcripts [5]. With the realization that RNA sequencing reads come from a non-uniform distribution and that reads can have positional and sequence specific biases, various improvements have been made in transcript abundance estimation [6]–[8]. Degradation can also have direct impact on transcript estimation and an RNA degradation model for RNA sequencing was recently published [9]. However, the direct effect of RNA degradation on differential expression and its correlation to the RIN has not, to our knowledge, been reported.

By fragmenting RNA prior to library preparation we systematically generate degraded RNA samples with a spectrum of RINs. A similar model of RNA degradation by fragmentation has been used before [10]. We show here that degraded samples, prepared with poly A selection, have 3' mapping bias and that the more degraded a sample is the more it deviates in differential expression from intact samples. On average, these degraded samples show an underrepresentation of long transcripts and an overrepresentation of short transcripts. Finally, we introduce a 3' tag counting (3TC) approach, and show that it reduces false positives in differential expression comparisons between samples of different quality.

## Materials and Methods

### 1. Sample preparation

U251 MG brain gliablastoma cells (Professor Bengt Westermark, Uppsala University) were cultured as described previously [11]. RNA was extracted from the cells using RNeasy kit (Qiagen). In total we obtained 64 g of RNA with RIN 10. 4 g were kept intact while the rest of the RNA was divided into 22 equal batches and each batch fragmented under different incubation time and temperature using the NEBNext Magnesium RNA Fragmentation Module. This fragmentation served as an emulator of degradation and resulted in RNA with a wide spectrum of RINs which we categorized into groups of RIN 2, 4, 6, 8 and 10. RNA profiles were evaluated and RIN calculated using 2100 BioAnalyzer (Agilent). From these RNA samples we prepared three libraries each of RIN 10, 8, 6, 4 and one library of RIN 2 using slightly altered and automated Illumina TruSeq library preparation protocol [12]; mRNA purification using poly-T oligonucleotides, mRNA fragmentation, first strand synthesis, second strand synthesis, carboxyl acid (CA) purification, end repair, adapter ligation, indexing, PCR amplification and CA purification. Additional four libraries; one of RIN 6, two of RIN 4 and one of RIN 2, were made using ribosomal depletion (RiboMinus kit, Invitrogen) instead of mRNA enrichment. Schematic overview of library preparation protocols and additional information can be found in Figure S1.

One of the RIN 8 sample failed in library preparation and was not sequenced. All the remaining 16 libraries were then clustered on cBot and sequenced on HiSeq 2000 according to manufacturer's instructions. Base conversion using OLB v1.9, demultiplexed and converted to fastq using CASAVA v1.8. This gave us 423 million 100 bp paired end reads that passed through Illumina's chastity filter, averaging to 26.4 million paired end reads per sample. All the raw reads have been submitted to the NCBI Sequence Read Archive under accession SRP023548 (SRA, http://www.ncbi.nlm.nih.gov/Traces/sra/).

### 2. Data analysis: Preprocessing/quality control

Before expression analysis the data were processed through a quality control pipeline consisting of three major steps: i) quality trimming and adapter removal, ii) mapping to a reference genome and iii) removal of ribosomal reads. For the quality and adapter removal the utility program Trim Galore! [6] was used. Trim Galore is a wrapper script that makes use of the trimming tool cutadapt [14]. Possible adapter sequences, based on the Illumina TruSeq Adapter index sequences, were removed from the reads. The reads were then quality trimmed, with a quality threshold of 30 on the Phred scale, and if either read from a pair was shorter than 30 bp after trimming that pair was removed from the analysis. The remaining *quality reads* were mapped to the GRCh37.68 primary assembly of the human genome (ensembl.org) using Tophat, version 2.0.2 [15]. When reads mapped to multiple locations only the primary hits as determined by Tophat were retained. Then, using the *split_bam.py* script from the quality control package RSeQC [16], the reads that mapped to ribosomal RNA locations in the genome were evaluated and removed from analysis. The remaining non-ribosomal reads, termed *useable reads*, were used for expression analysis.

Additionally, the amount of duplicate reads were quantified using MarkDuplicates from Picard [17]. However, since duplicate reads can be wrongly called, especially for highly expressed genes, these reads were retained in the downstream differential expression analysis.

### 3. Data analysis: Differential expression

Htseq-count [18] was used to count how many reads match to each feature in an annotation file. The annotation file is in gene transfer format (GTF) and a feature is, in our case, an ensembl gene ID. We set the -m parameter to *intersection-strict*. All genes then went through custom filtering which is defined as follows: If the average read count of a gene across the sample groups was below five counts then that gene was removed from analysis due to low expression; all other genes were retained in the analysis and are called as expressed genes. This filtering step was included to try and reduce false positives in the differential expression [19]. The R package DESeq was used to get differential expression between sample groups [20]. The DESeq package normalizes for different sample read depths and is able to carry out differential expression analysis even if one of the groups in the comparison only contains one sample, which is why the RIN 2 group is included in the analysis even though it has no replicates. All genes with a p-value below 0.05 after Benjamini-Hochberg adjustment (FDR) were labeled as differentially expressed genes, hereafter referred to as DEGs. We limited our differential expression analysis to protein coding genes.

### 4. Data analysis: 3' tag counting

**Figure 1:**
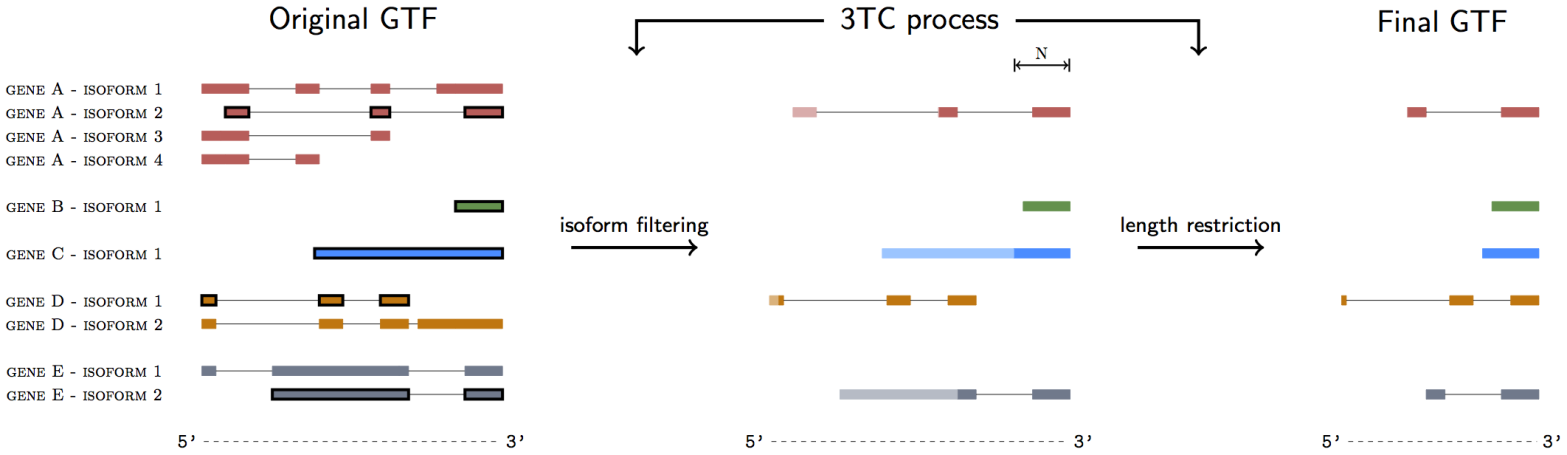
Schematic drawing of the 3TC method. Shown is a graphical representation of the annotation file used for counting. The original annotation file contains all annotated exons and isoforms for all genes and is shown on the left. The isoforms with the highest expression within a gene are indicated by a black borderline around its exons. The two steps of the 3TC process are shown in the middle; only those isoforms that have the highest expression are kept in the annotation file (isoform filtering) and all isoforms are truncated to a specific length N (length restriction, shown by shading). The final annotation file is shown on the right.

Many false positive DEGs arise due to the difference in RIN between experimental groups. To correct for this false positive rate we restricted the counting to those reads that mapped to the 3' end of the genes, a counting method we call 3' tag counting (3TC). A schematic diagram explaining the basic features of our 3TC counting method is shown in Figure 1. The 3TC method involves changing the GTF annotation file, that is used when counting, in two steps; i) isoform filtering and ii) transcript length restriction. The isoform filtering step selects which isoform of each gene to retain as to prevent count contamination from overlapping isoforms. The transcript length restriction limits each isoform in the annotation file to a certain length N, counting from the 3' end, and all bases and exons beyond that length are left out in the counting process. This is done because the gene coverage of the 5' end diminishes the more the sample is degraded while the coverage closer to the 3' end is greater, regardless of sample quality.

Deciding which isoforms to retain and which isoforms to filter out is not straightforward but we opted to retain the isoform which shows the highest expression within a gene. To determine this highest expressed isoform, the isoform FPKM value from the RIN 10 group was calculated using Cufflinks [21]. The isoform which had the highest expression within a gene was retained in the annotation file. If none of the samples are of high quality we provide an alternative approach to isoform filtering, shown in Figure S2, which is independent of the samples used but of somewhat lower sensitivity.

Applying the 3TC method with length restriction reduces the number of genes labeled as expressed, i.e. it decreases sensitivity. We thus evaluate the success of the method as a function of sensitivity which is defined as: **sensitivity**  =  (no. of genes called as expressed, using N = X)/(no. of genes called as expressed, using N = 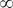); where N is the length restriction of the 3TC and X, in our case, is 1500, 1000, 500 and 200 nt (see Table 2).

For a negative control for the effects of the 3TC method, we used previously published libraries from the U2OS and U251 cell lines [20]. These libraries are from high quality RNA and the 3TC method should not have substantial effect on their differential expression. Also, as a second control we checked what effects the 3TC method had on differential expression between replicates of the same quality. Since there were, at most, only three replicates, denoted R1, R2 and R3, within group this analysis does not have substantial power. Nonetheless, for the groups RIN 10 and RIN 6, we performed differential expression analysis by comparing R1 vs. R2+R3, R2 vs. R1+R3 and R3 vs. R1+R2.

## Results

### 1. Degradation of RNA

**Figure 2:**
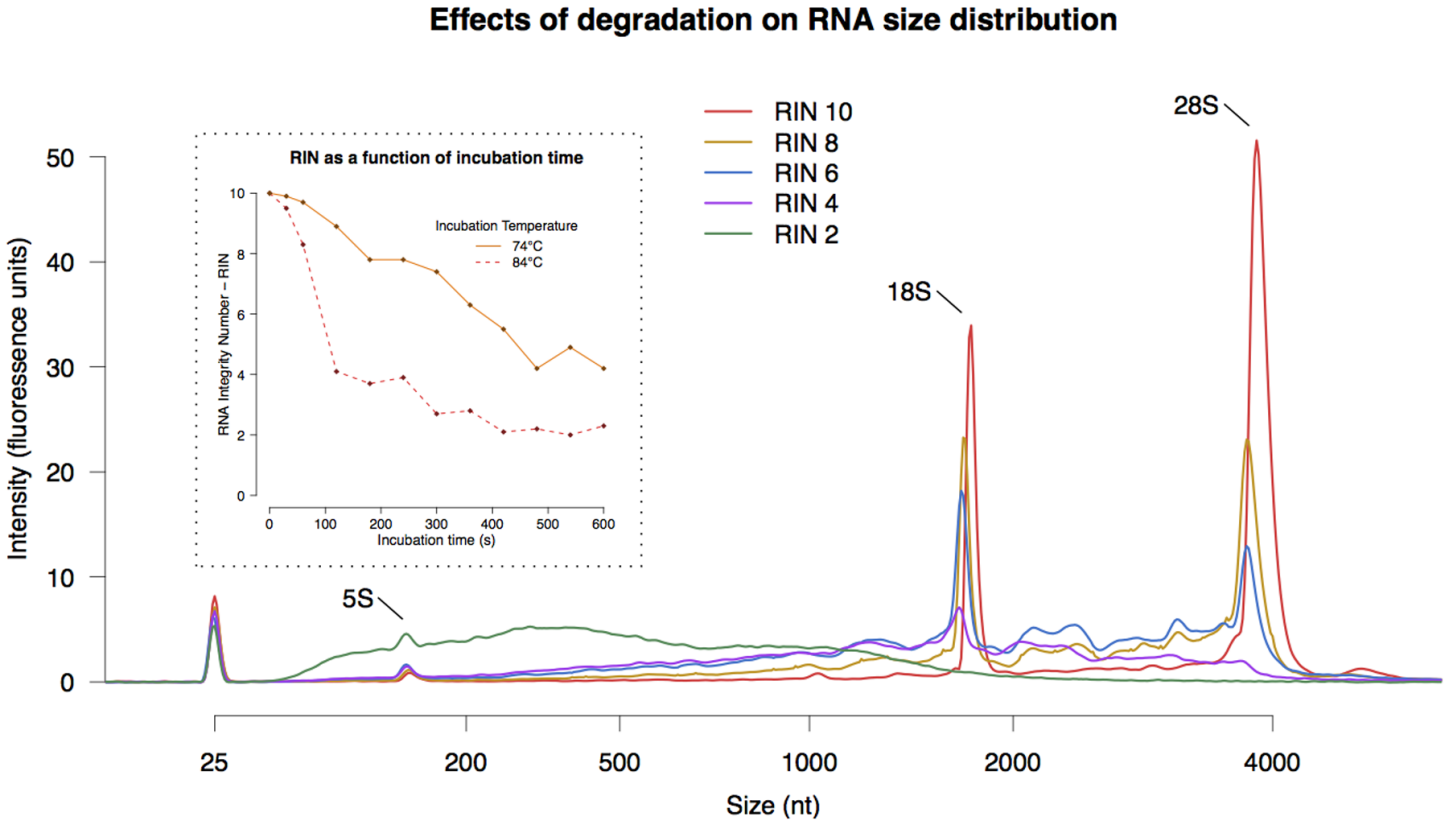
Effects of degradation on the RNA size distribution for different RINs. The large ribosomal RNA peaks, 18S and 28S, show a steady decrease with decreasing RIN and completely disappear for RIN 2. Also apparent is an increase of small molecules with decreasing RIN. This is especially noticable for the RIN 2 sample. The dotted inset shows how the magnesium ions affect the RIN of the RNA as a function of temperature and incubation time.

Incubation of intact RNA in magnesium ion solution, for different time periods and at different temperatures, results in RNA with a spectrum of RIN (see inset in Figure 2). The RIN is dependent on the ribosomal peaks (18S and 28S) of the RNA and they progressively diminish the more the samples are degraded (see Figure 2).

**Figure 3:**
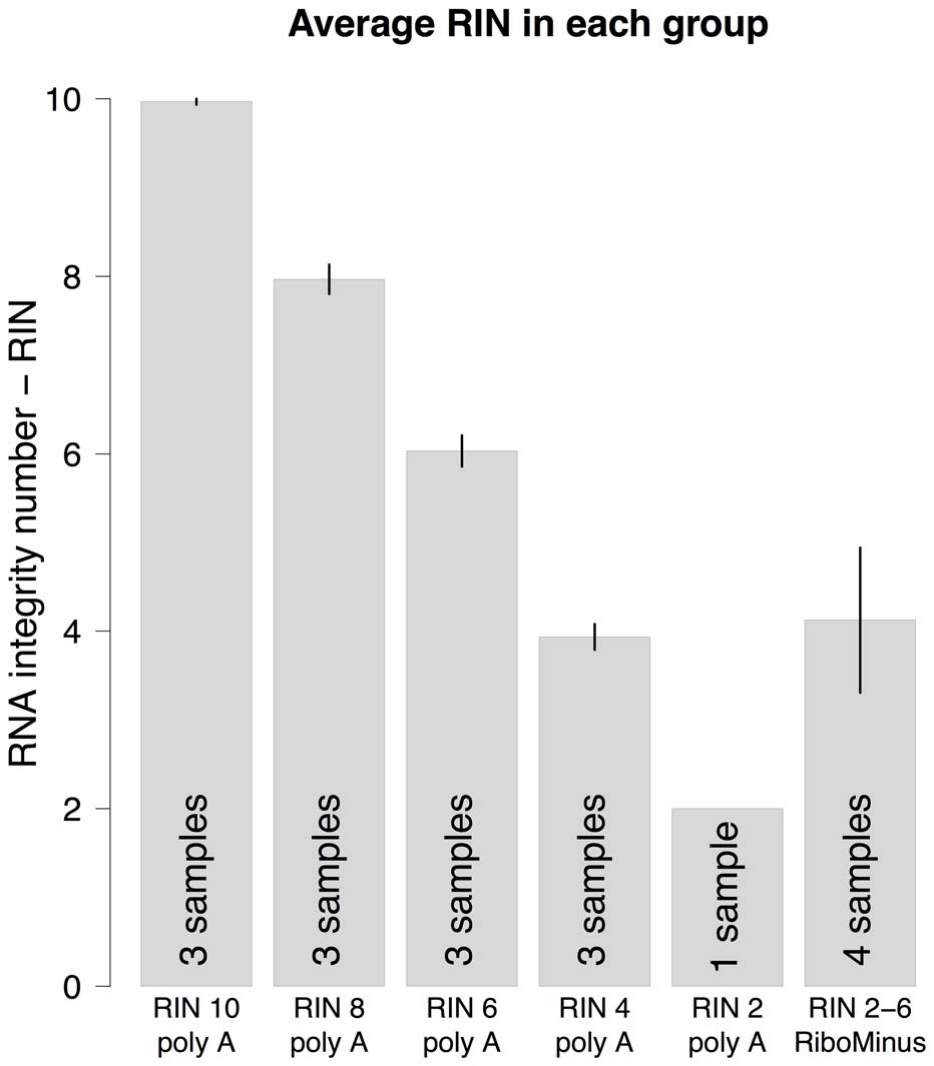
Attributes of the experimental groups. The height of the bars represents the average RIN for each group along with error bars. The bottom of each bar shows the number of samples in each group. Below each bar are the group names which both show the average RIN as well as the enrichment method used for the samples in the groups. Error bars denote the standard error. One of the RIN 8 samples failed in library preparation thus for the subsequent sequencing data there are only two samples in the RIN 8 group.

From the degraded RNA, six experimental groups were prepared; RIN 10, RIN 8, RIN 6, RIN 4, RIN 2 and RiboMinus (RIN 2–6). Each group contained RNA from three samples except the groups RIN 2 and RiboMinus which consisted of one and four samples respectively. The barplot in Figure 3 summarizes information of the experimental groups.

### 2. Preprocessing of sequencing data

**Figure 4:**
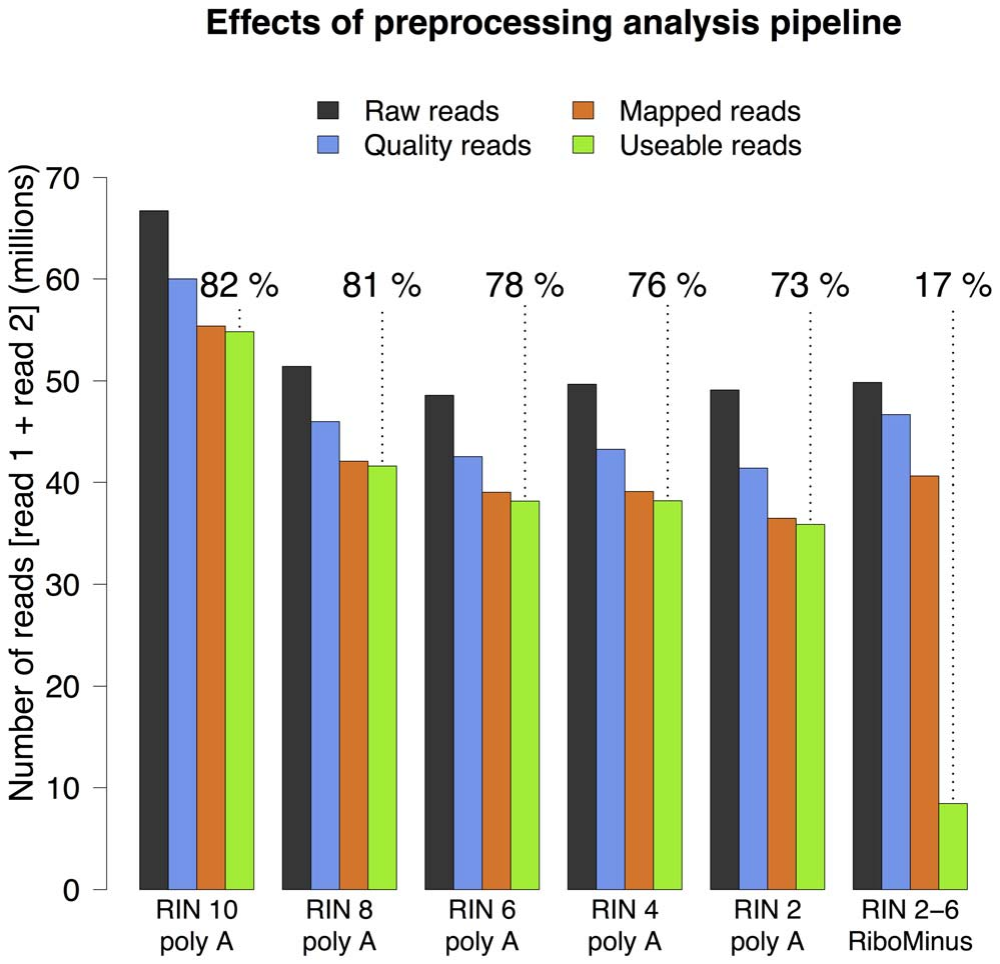
Preprocessing of sequencing data. The barplot shows how many reads survive through each of the steps of the preprocessing pipline (see Methods). The step of going from Mapped reads to Useable reads is removal of rRNA. A large amount of reads are lost due to rRNA read removal in the RiboMinus group. The pecentage of useable reads (shown above the dotted lines) shows a steady decline with decreasing RIN. The poor performance of the RiboMinus samples can be attributed to high rRNA contamination.

A detailed overview of the effects of the preprocessing pipeline (see Methods) on each of the individual libraries is shown in Table S1 and summarized for the experimental groups in Figure 4; with the percentage of useable reads for each group shown above the *Useable reads* bar. A noticeable decline in useable reads is observed with decreasing RIN, an effect that accumulates thorugh the steps of the preprocessing pipeline. A one-way ANOVA finds a statistically significant difference in useable reads between the RIN groups (F = 4.9, p
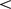
0.05) and a Tukey HSD test reveals that the statistical difference is only between the RIN 10 and the RIN 2 groups (p
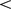
0.05). As shown in Table S1 and Figure S3 there is a noticeable increase in duplicate reads with decreasing RIN and the difference in remaining reads after duplicate removal becomes more prominent (see Figure S3 for statistical details). In the downstream differential expression analysis the duplicate reads were retained.

The strikingly poor performance of the samples treated with the RiboMinus kit is predominantly due to high ribosomal RNA (rRNA) contamination in the mapped reads; for the samples in this group 79% of the reads map to ribosomal RNA genes, on average. Thus the RiboMinus kit effectively fails to remove ribosomal RNA from the degraded samples.

### 3. Gene body coverage

**Figure 5:**
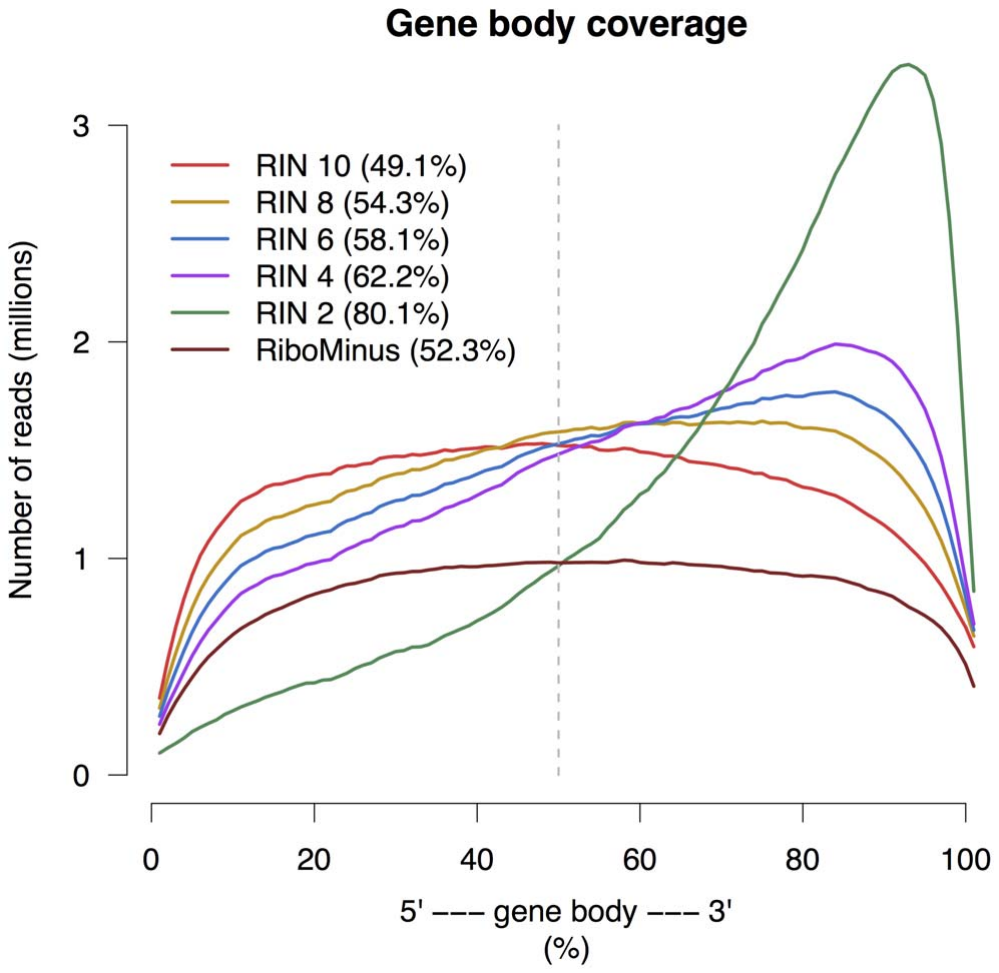
Gene body coverage on average for each group. Both RIN 10 and RiboMinus show even coverage. The percentages in the paranthesis show the relative amount of reads that map closer to the 3' end than to the 5' end, i.e. the amount of reads that map to the right of the dashed vertical line. Each step of decreasing RIN shows an increase in 3' bias.

In order to make a reliable comparison between the groups, which were of different sequencing depth, the reads from each library were downsampled to 6 million reads using Picard tools [17]. The gene coverage over the entire gene body for each group is visulized in Figure 5. There is an increased 3' mapping bias with decreasing RIN. This mapping bias formed the basis for the 3' tag counting approach used for normalization in differential expression analysis (see Methods and below). Despite the massive loss of reads in the RiboMinus group it does show an even gene coverage.

Figure 5 is readily explained on the basis of the enrichment method used in the sample preparation step. The standard poly-A selection is a form of positive selection (i.e. selects molecules to keep in analysis) so if the sample is degraded the data will reflect that. Ribosomal depletion, however, is a form of negative selection (i.e. selects molecules to remove from analysis) so even if a sample is degraded the data should retain even coverage, because fragments all along the RNA will be collected rather than just those containing the 3' end used for polyA selection. Even though all libraries were downsampled to include equal amount of reads the RiboMinus group has fewer reads along the gene body; this is because the RiboMinus group has a higher proportion of reads mapping to unannotated regions.

### 4. Effects of degradation on differential expression

In order to make a reliable comparison between the groups the reads from each library were downsampled to 20 million reads using Picard tools [17] except when making a comparison to the RiboMinus group in which case the libraries were downsampled to 6 million reads.

Many DEGs arise between the experimental groups solely because of different degradation of the samples. For example when comparing RIN 10 to RIN 8, 4377 genes are differentially expressed or around 36% of all the expressed genes. When comparing RIN 8 to RIN 6, however, only 109 (1%) genes show up as differentially expressed. Table 1 shows the number of DEGs between other groups.

**Table 1:** Effects of degradation on differential expression.

Comparison	No 3TC	3TC; N = 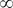	Shared
RIN 10 vs. RIN 8	4377 (36%)	4344 (36%)	4133
RIN 10 vs. RIN 6	6827 (56%)	6841 (56%)	6501
RIN 10 vs. RIN 4	6886 (56%)	6897 (57%)	6551
RIN 10 vs. RIN 2	6664 (54%)	6795 (56%)	6319
RIN 10 vs. RM 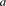	3943 (33%)	3778 (32%)	3651
RIN 8 vs. RIN 6	109 (1%)	125 (1%)	99
RIN 8 vs. RIN 4	1500 (12%)	1583 (13%)	1396
RIN 8 vs. RIN 2	2663 (22%)	2955 (24%)	2498
RIN 6 vs. RIN 4	270 (2%)	310 (3%)	256
RIN 6 vs. RIN 2	2382 (19%)	2661 (22%)	2214
RIN 4 vs. RIN 2	561 (5%)	764 (6%)	510
Control 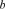	10484 (76%)	10263 (75%)	10097

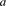
RM  =  The RiboMinus group (RIN 2–6).

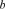
Control  =  Two different cell lines (U2-OS and U251) from high quality RNA [20].

Number of DEGs without 3TC method and with 3TC (isoform filtering only, no length restriction). The first column shows the comparison, the second column shows the number of DEGs without 3TC, the third column shows the number of DEGs with isoform filtering and the forth column shows the number of DEGs that are shared between the two methods. The percentage in parenthesis is the percentage of the total number of genes that are labeled as expressed. See also Figure S4.

To determine the effect of the isoform filtering step of the 3TC method has, in general, on differential expression the reads were counted towards an annotation file that had been processed through the isoform filtering step of 3TC but no length restriction (effectively the 3TC method with N = 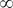, see Figure 1). The number of DEGs compare very well between the two methods (the two methods being *no 3TC* and *3TC with N =* 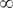). Table 1 lists the number DEGs for the two methods as well as the number of DEGs that are shared between them. Figure S4 shows graphical representation of Table 1 using Venn diagrams.

**Figure 6:**
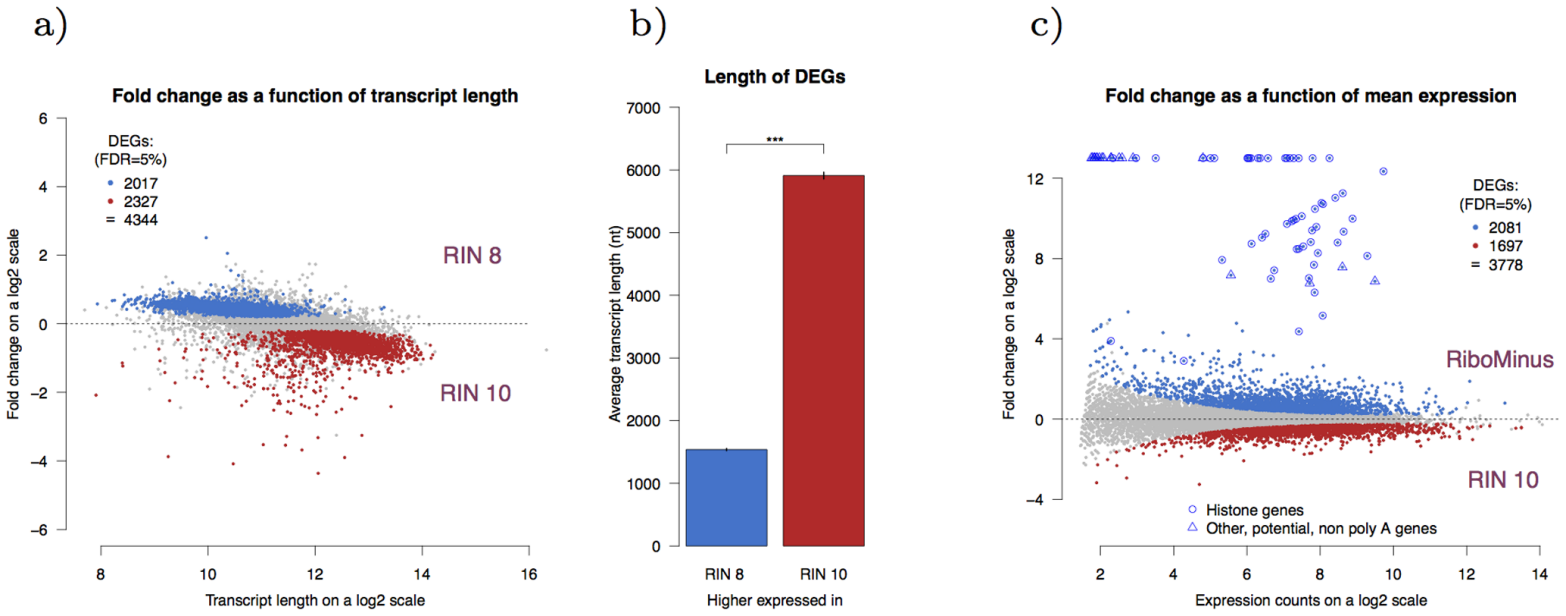
Differential expression of degraded RNA. (**a**) A common feature of the differential expression profiles is that long transcripts tend to be more highly expressed in the group with higher RIN and, reversely, short transcripts tend to be more highly expressed in the group with lower RIN. Shown here is the expression profile for the comparison RIN 10 vs. RIN 8, with log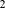 of the fold change (fold change  =  expr(RIN 8)/expr(RIN 10)) on the y-axis and transcript length on the x-axis. (**b**) The DEGs shown in (a) are split into two groups; the ones that have higher expression in RIN 10 (red) and the ones that have higher expression in RIN 8 (blue). The average transcript length in the RIN 10 group is significantly higher than the average transcript length in the RIN 8 group (Student's t-test, p
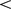
0.001). Error bars denote the standard error. The distribution of these gene lengths is shown in Figure S6. (**c**) Expression profile of the comparison RIN 10 vs. RiboMinus. In total there are 3778 DEGs; with 2081 upregulated in the RM group and 1697 upregulated in the RIN 10 group. Some of the genes upregulated in the RM group show markedly high fold change. Many of those, marked with a circle, are histone genes. The transcripts of histone genes lack a poly A tail which explains why they show a markedly higher expression in the samples prepared with ribosomal depletion compared to samples prepared with poly A selection. Additionally, genes that show similar trend have been marked with a triangle. These data indicate that those genes may lack or have repressed poly adenylation sites.

A common feature of these comparisons is that DEGs that have higher expression in the group with higher RIN are, on average, longer than the DEGs that have higher expression in the group with lower RIN. The average length of the selected transcript isoform of the 2017 DEGs that have higher expression in RIN 8 is 1500 nt while the corresponding average length of the 2327 DEGs that have higher expression in RIN 10 is 5900 nt. This length difference is significant (p
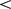
0.001, Student's t-test). This is illustrated in Figure 6a and Figure 6b for the comparison RIN 10 vs. RIN 8 and in Figure S5 for the rest of the comparisons.

Interestingly, even though the RiboMinus group consists of samples of low quality RNA (RIN 2–6) only 3778 DEGs are found when comparing it to the RIN 10 group, which is considerably less than the 4344 DEGs found when comparing RIN 10 to RIN 8. This is attributable to the enrichment method used for the RiboMinus group which gave rise to the even gene body coverage for that group as discussed above.

The expression profile for the comparison RIN 10 vs. RiboMinus, displayed in Figure 6c, identifies genes with much higher expression in the RiboMinus group compared to the RIN 10 group. Many of those genes turn out to belong to histone genes but histone gene transcripts are known to be without poly A tail [23]. This led to the conclusion that more genes that have distinctively higher expression in the RiboMinus group compared to the RIN 10 group may be genes without, or repressed, poly adenylation sites. In total there are 18 potentially poly A (-) genes which are listed in Table S2.

**Figure 7:**
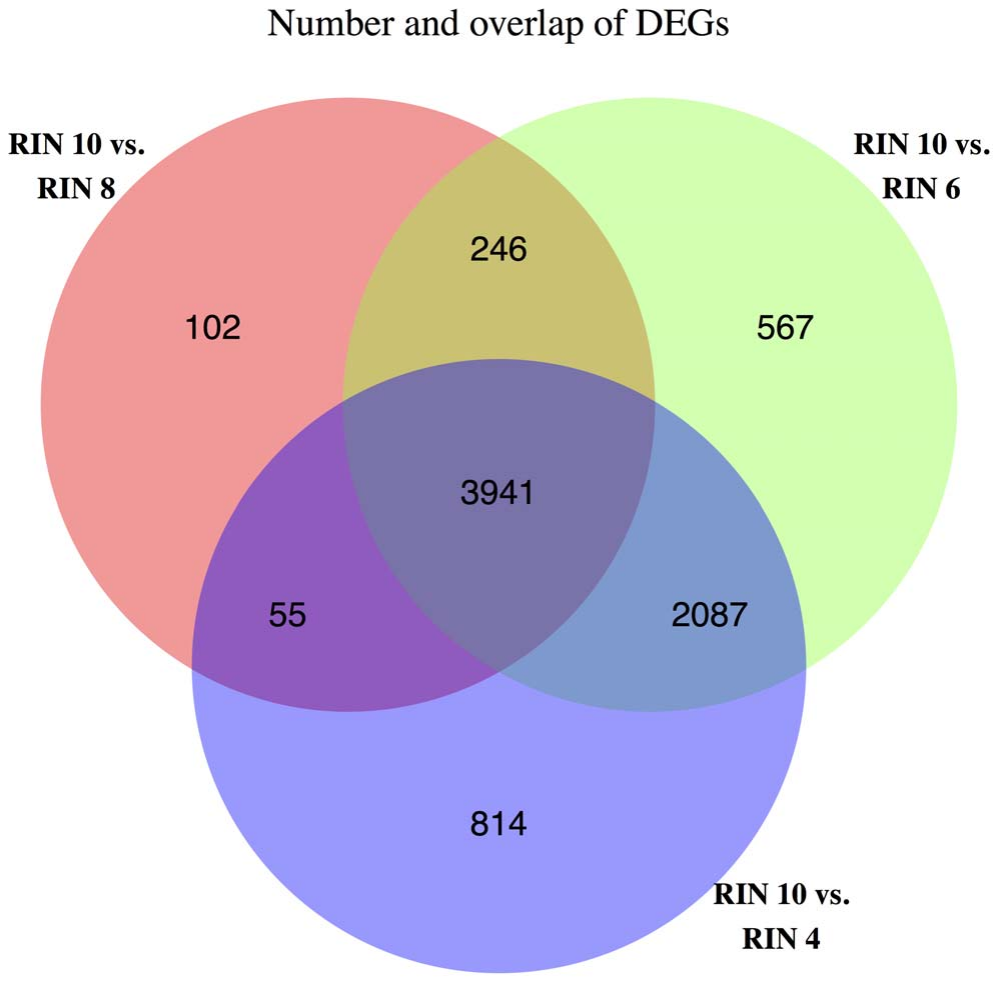
Overlap of DEGs between the first three comparisons from Table 1. Majority of the DEGs found in the comparison RIN 10 vs. RIN 8 are also found in the other two comparisons. While there is not an increase in DEGs when comparing RIN 10 to RIN 6 and RIN 4 the overlap between those two comparisons are considerable.

The Venn diagrams in Figure 7 show the overlap of the DEGs found in the first three comparisons in Table 1. The overlap shows that the increase in DEGs is mainly additive, i.e. the DEGs found in the comparison RIN 10 vs. RIN 8 are also found in the comparison RIN 10 vs. RIN 6 and RIN 10 vs. RIN 4.

### 5. Effects of 3TC for differential expression

The previous section demonstrated that DEGs arise between different RIN groups and that isoform filtering, the first step in 3TC, only has minimal effect on the results of differential expression. Applying 3TC using varying constants for the maximum isoform length, N: 1500 nt, 1000 nt, 500 nt and 200 nt, realizes the full potential of the method.

**Figure 8:**
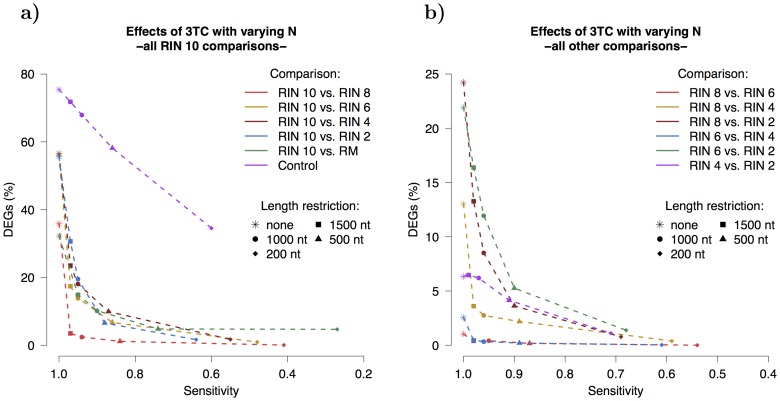
Effects of 3TC method on differential expression. The y-axis shows the percentage of DEGs and the x-axis shows the sensitivity. The colors denote different comparisons while different shapes of points denote the varying N used in the length restriction process. (**a**) All RIN 10 comparisons. All comparisons demonstrate a sharp decrease in DEGs going from no length restriction to N = 1500 nt. Lowering N further results in fewer DEGs but at the expense of sensitivity. The control, which compares two different cell lines, does not show any abrupt decrease in DEGs but rather follows a straight line. (**b**) All non-RIN10 comparisons. All comparisons, except RIN 4 vs. RIN 2, show improvement with the 3TC (N = 1500) method. This is even true for the two comparisons, RIN 8 vs. RIN 6 and RIN 6 vs. RIN 4, where originally there were very few DEGs. [**DEGs**  =  differentially expressed genes].

There were 4344 DEGs for the comparison RIN 10 vs. RIN 8 without any length restriction. The number of DEGs decreases to 413, 278, 116 and 2 for N = 1500, 1000, 500 and 200 nt, respectively. This improvement does come at a cost of decreasing sensitivity but the sensitivity remains remarkably high until the 200 nt length restriction (sensitivity is the ability of the method to call genes as expressed, see Methods for definition of sensitivity). The details of the 3TC results for the comparison RIN 10 vs. RIN 8 is shown in Table 2 and for all other comparisons in Table S3. A graphical overview of how the comparisons fared in the 3TC method is shown in Figure 8a for all RIN 10 comparisons and Figure 8b for all other comparisons. The control comparison, from high quality RNA of two different cell lines, does not show the substantial decrease in DEGs as the other groups. Also the 3TC method does not seem to have a significant effect on differential expression within groups but for the RIN 10 group the number of DEGs between replicates were, on average: 2, 2, 1, 3, 1 for N = 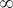, 1500, 1000, 500, 200 respectively. Similarly, for the RIN 6 group the number of DEGs were, on average: 17, 12, 7, 3, 0 for N = 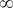, 1500, 1000, 500, 200 respectively.

**Table 2:** Details of the 3TC method performance for the comparisons RIN 10 vs. RIN 8.

Length restriction	Expressed genes	DEGs	DEGs (%)	Sensitivity
none	12212	4344	35.8%	1.00
1500	11827	413	3.5%	0.97
1000	11458	278	2.4%	0.94
500	10210	116	1.1%	0.84
200	5065	2	0.0%	0.41

By setting the length restriction to 1500 nt the percentage of DEGs go from 35.8% down to 3.5% with little loss of sensitivity, from 1.00 to 0.97. When the length restriction is set to 200 nt the loss of sensitivity becomes considerable. Details of the other comparisons are found in Table S2.

**Figure 9:**
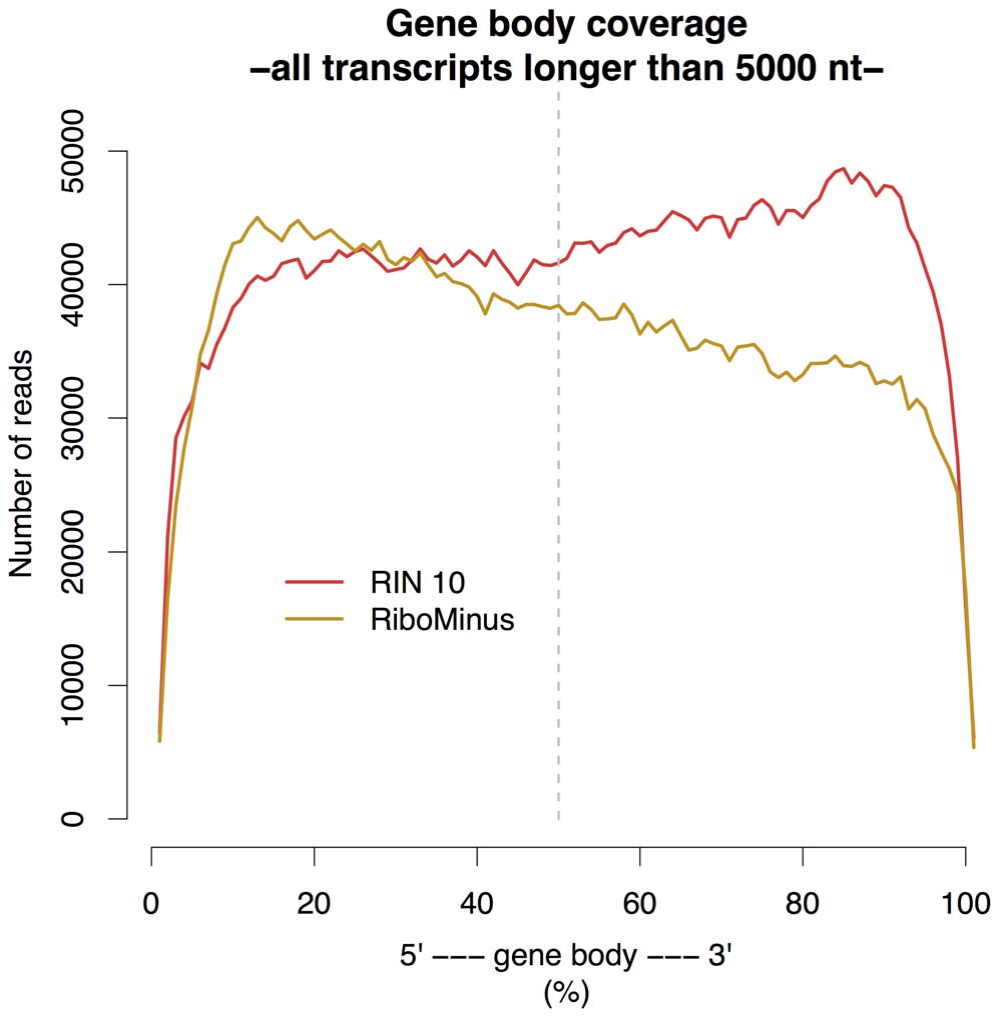
Gene body coverage for transcripts longer than 5000 nt. For transcripts longer than 5000Figure 5. These biases may explain why the 3TC method decreases the number of false positives found in the RIN 10 vs RM comparison as shown in Figure 8a.

Again worthy of attention is the RiboMinus group which, despite showing even gene body coverage, shows improvement using the 3TC method; from 3778 DEGs with 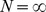 to 1661 DEGs using 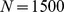 nt, in the comparison RIN 10 vs. RiboMinus. Also in this comparison, contradictory to all the other comparisons, the DEGs overrepresented in the RiboMinus group are longer than the DEGs overrepresented in the RIN 10 group (see Figure S5). An explanation for this behaviour may lie in the fact that even if these two groups, RIN10 and RiboMinus, show even gene coverage on average the gene coverage is not even for the genes with the largest transcripts. In fact, as shown in Figure 9, for large transcripts the RIN 10 group has a three prime bias wheras the RiboMinus group has a five prime bias.

### 6. Effects of Cufflinks bias correction on differential expression

Of the methods mentioned (see Introduction) that aim to improve transcript estimation by assuming non-uniform read distribution, or take RNA degradation into account [6], [7], [9], only the bias correction by Roberts et. al. [8] offers a direct downstream analysis of differential expression through Cuffdiff. Table 3 shows the number of differentially expressed genes with and without using the bias correction from [8]. In all comparisons the bias correction worsens the outcome of the differential expression.

**Table 3:** Number and percentage of DEGs as found by Cuffdiff with and without bias correction.

Comparison	No bias correction	Bias correction
RIN 10 vs. RIN 8	2266 (20%)	2962 (26%)
RIN 10 vs. RIN 6	4185 (37%)	4724 (42%)
RIN 10 vs. RIN 4	4471 (39%)	5130 (45%)
RIN 10 vs. RIN 2	6845 (60%)	8494 (73%)
RIN 8 vs. RIN 6	209 (2%)	293 (3%)
RIN 8 vs. RIN 4	1021 (9%)	1183 (11%)
RIN 8 vs. RIN 2	4221 (37%)	7127 (63%)
RIN 6 vs. RIN 4	203 (2%)	242 (2%)
RIN 6 vs. RIN 2	3809 (34%)	7232 (64%)
RIN 4 vs. RIN 2	1944 (17%)	6093 (55%)

In all comparisons the bias correction increases the instances of false positives.

## Discussion

### 1. Systematic generation of degraded RNA by fragmentation

RNA degradation in the cell is carried out by endonucleases and exonucleases. The endonucleases break the phosphodiester bonds within the RNA fragment while exonucleases degrade the RNA from either end. RNA degradation can also occur during RNA extraction and RNA handling by other means, such as physical shearing. We fragmented intact RNA with magnesium ions prior to library preparation to achieve degraded RNA as estimated by the RIN.

While our degradation method does not reflect any exonuclease activity the data gives us no reason to doubt it emulates *in vitro* degradation in other respects. Indeed it has been argued previously that RNA degradation is random and RNA degradation models are based on that assumption [10].

### 2. Sequencing degraded RNA: consequences

There are some issues to be aware of when sequencing degraded RNA. By following standard fragmentation procedures during library preparation a degraded sample will result in lower complexity libraries and thus higher duplication rates. This loss in complexity may possibly be prevented, at least partly, by carefully controlling the fragmentation step during libary preparation but this, however, was not investigated here. Degradation also leads to a loss of full length transcript as shown by the 3' mapping bias which can give rise to false positives in differential expression analysis. Furthermore, long transcripts tend to be overrepresented in samples of higher quality when compared to samples of lower quality where short transcripts are overrepresented (Figure 6).

Sequencing protocols often set a certain quality threshold for RNA samples (RIN
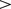
7 or 8) to be processed into libraries. According to our results there does not seem to be any justification to set a threshold at any specific RIN but rather it is important to be aware of the effects of low RIN and all samples should preferably be in close range in terms of quality. Indeed, the only relevant RIN threshold the data suggests is at RIN 10 since the most dramatic effect in differential expression is when samples deviate from RIN 10, i.e. we achieve far more DEGs when comparing RIN 10 vs. RIN 8 than when comparing RIN 8 vs. RIN 6.

### 3. Sequencing degraded RNA: solutions

The 3TC approach we present here is both simple and straighforward and manages to reduce the number DEGs between samples of different quality. As such it could be useful for researchers that work with data from degraded RNA samples. Which value to set to the length restriction, N, is speculative but N = 1500 usually shows marked decrease in false positives while maintaining high sensitivity (sensitivity is the ability of the method to call genes as expressed, see Methods for definition of sensitivity) and can be considered as a safe upper limit. Arguably, even working with relatively good quality RNA, using the 3TC method for differential expression could be a valid option to decrease false positives. If analyzing samples where there is a significant difference in quality between the samples, lowering N below 1500 might be neccessary despite the loss of sensitivity.

The main drawbacks of the 3TC approach is that it is gene based, i.e. it is not suitable for alternative splicing analysis, and the genes are represented only by their major isoform. The fact that it is gene based may introduce some false negatives in differential expression analysis since a gene can express different isoforms at different levels between different conditions. However, this is only of concern when dealing with expressed isoforms other than the major isoform. If there is an expression difference between major isoforms of genes it should be picked up by the 3TC method. It is noted that the major isoform may not be the ideal representative for a gene, however it has been shown that the major isoform of a gene with five or less variants usually represents more than 60% of the gene's transcript abundance and similarly for genes with 5–15 isoforms the major isoform represents more than 40% of the gene's transcript abundance [24]. While not perfect this is an indicator that the major isoform can be a decent proxy for the whole gene and if the more abundant isoforms of a gene share many of their exons it makes this case even stronger. That being said, there may be situations where the 3TC method, using the major isoform as a proxy for the gene expression, is unapplicable.

Another solution when working with degraded RNA is to use ribosomal depletion for enrichment instead of poly-A selection. In our case we used the RiboMinus kit and those samples perform better in gene coverage and differential expression. The RiboMinus kit however failed in removing rRNA from degraded RNA samples and cannot be claimed as a good option for working with degraded RNA. While the poor performance of the RiboMinus kit can partly be explained by the fact that it only contains two rRNA probes and as such it is not suitable to work with degraded RNA we do have data showing slightly improved, but still unacceptable, performance of the RiboMinus kit with high quality RNA (see Figure S7). Since performing these experiments the RiboMinus kit has been taken off the market and is now superseded with the RiboMinus kit v2. Indeed, improved ribosomal depletion kits are continuously developed which supposedly are more suitable for preparing RNA samples of low quality. A recent paper, which benchmarks five different protocols for low quality and/or low quantity samples for RNA sequencing, reports rRNA read mapping from 0.1% (most efficient protocol, RNase H) to 23.2% (least efficient protocol, NuGen) [1]. Any of these methods is presumably an efficacious option when working with RNA samples with low RIN.

These depletion methods for total RNA sequencing are sequence dependent and are not available for all species and, apart from the RNase H method [14], require relatively high amount of starting material. The standard enrichment method of poly A selection is the one most readily available and most frequently used and can be carried out using low amount of starting material.

Finally, it should be emphasized that the majority of all archived RNA sequence data to date is derived from poly A selection. Accordingly, our 3TC method is pertinent to contemporary RNA sequence analysis as well as for reevaluation of archived RNA sequence data.

### 4. Alternative isoform selection in 3TC

As mentioned in the Methods section, our approach for the isoform filtering step, i.e. selecting the most highly expressed isoform in the group with the highest RIN, may, for one reason or another, not always be applicable. In Figure S2 we show an alternative approach for the isoform filtering step which is solely dependent on the annotation file and not on the expression on any of the samples used. For comparison we include the results using this alternative isoform filtering approach in Figure S8 and on Table S4. In broad strokes the second approach shows similar results albeit with a slight decrease in sensitivity. This decrease in sensitivity is likely due to alternative polyadenylation sites for different isoforms [26], [27] so that sometimes the most highly expressed isoform does not contain the exon (or exons) most downstream on a gene.

### 5. Comparison to other methods

We performed differential expression analysis on degraded samples using both the 3TC method and the Cufflinks bias correction. The 3TC method decreases the number of false positives substantially while the Cufflinks bias correction actually performs worse than Cufflinks without bias correction. It should, however, be stated that the Cufflinks bias correction was not directly designed to model RNA degradation but it still raises some concern to the validity of the module. While there have been other published methods on improving transcript estimation they do not seem to offer any direct downstream analysis for differential expression and thus were not compared to our 3TC method.

There exist interesting laboratory protocols, in the context of 3TC, which show promise or have potential in working with low quality RNA samples[26]–[30]. These protocols work by sequencing only the portion of the transcripts close to the polyadenylation sites and involve fragmenting good RNA samples prior to poly A enrichment, effectively probing the 3' ends of the transcripts. Working only with 3' ends, comparison between high and low quality samples becomes feasible. These protocols are in some ways comparable to our 3TC method, however by using the 3TC method there is no need to intentionally fragment quality samples or change the library preparation protocol. Also, the 3TC method is scalable through the length restriction step.

Finally, the authors of the PAS-Seq mehtod identify a list of 22 poly adenylated histone genes[26]. Surprisingly 19 of these 22 genes show up as significanly differentially expressed in our RIN 10 vs RiboMinus comparison indicating that they are *not* poly adenylated. The other three genes (HIST1H4J, HIST1H4K and HIST2H4A) show up as unexpressed in both groups.

## Supporting Information

Figure S1 **Flowchart of the two library preparations protocols used.** **a**) poly-A enrichment and **b**) ribosomal depletion. The steps shaded with blue have been automated on a Magnatrix 1200 Biomagnetic Workstation. After RNA extraction the RNA is run through a quality check (QC) with a BioAnalyzer and Qubit quantification fluorometer. The dotted QC line indicates that it is only safe to continue with the procedure depending on the results from the quality check. The cDNA libraries go through the same quality check procedure after the CA purification (The CA purification is a washing step based on carboxil acid beads). The alterations from the standard protocol lie in the automation and the CA purification as well as the ribosomal depletion for the protocol in b).
(PDF)

Figure S2 **Schematic diagram of the alternative isoform filtering for 3TC.** Alternative isoform filtering for 3TC, independent of samples used. Instead of determining the highest expressed isoform, within a gene, from the group with the highest quality, we select the isoforms that are closest to the three prime end. This approach is simpler in the sense that here it is not necessary to determine the highest expressed isoform within a gene and it is independent of the expression of the samples used. However it does not perform as well since it will, occasionally, select isoforms that are lowly or not expressed. For results using this isform filtering approach, see Figure S8.
(PDF)

Figure S3
**Preprocessing of sequencing data with duplicate removal.** The barplot shows how many reads survive through each of the steps of the preprocessing pipline, as Figure 4 in the main text, with an additonal step of removing duplicates - from Non rRNA read to Useable reads. A large amount of reads are lost due to rRNA read removal in the RiboMinus group. The percentage of useable reads (shown above the dotted lines) shows a steady decline with decreasing RIN. This is a cumulative effect of each step but is mostly due to increasing amount of duplicates with lower RIN. A one-way ANOVA finds a statistically significant difference in useable reads between the RIN groups (F = 64.4, p
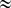
0.00001) and a Tukey HSD test reveals a statisitical difference between all groups (p
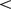
0.05) except between the RIN 10 and RIN 8 groups and between the RIN 8 and RIN 6 groups. The poor performance of the RiboMinus samples can be attributed to high rRNA contamination.(PDF)

Figure S4 **Venn diagrams showing the effects of isoform filetering on differential expression.** The number and overlap of DEGs between two methods counting for differential expression. One method 'No 3TC' (light green) counts towards an unaltered gtf annotation file as downloaded from ensembl.org. The other method '3TC - isoform filtering' (light red) uses an annotation file that has gone through the isoform filtering step of the 3TC method but no length restriction. In general there is good agreement with the two methods but the '3TC - isoform filtering' usually has more unique DEGs than the 'No 3TC' method. (These Venn diagrams are effectively visualizations of Table 1 from the main text.).
(PDF)

Figure S5 **Differential expression profiles between the experimental groups.** In almost all instances the DEGs that are more highly expressed in the group with higher RIN are, on average, significantly longer than the DEGs that are more highly expressed in the group with lower RIN. The two exceptions are the comparison between RIN 10 and RiboMinus where it is the opposite and the control group where there is no difference in the length of DEGs.
(PDF)

Figure S6 **The length distribution of up and down regulated DEGs from the comparison RIN 10 vs. RIN 8.** The dotted vertical lines show the means depicted in Figure 6b.
(PDF)

Figure S7
**Effects of RiboMinus of low and high quality samples.** Ribosomal RNA in samples with low and high RIN values after being treated with the RiboMinus kit. The samples in the Low RIN bar are the same RiboMinus samples used in the current study. The sampels representing the High RIN are from another unpuplished study using the same ribosomal depletion method. The Low RIN bar is the average from four samples while the High RIN bar is an average from six samples. The error bars show the standard error. The RiboMinus kit performs signficantly better on high quality samples (p
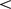
0.05, Student's t-test) but the High RIN samples still contain above 65% of rRNA reads which is must be considered unacceptable.(PDF)

Figure S8 **Comparison between two versions of the isoform filtering step of the 3TC method.** **a**) and **b**) is a reproduction of Figure 8 from the main text. **c**) and **d**) show the results using the alternative isoform selection explained in Figure S2. The versions show similar results but scrutinization reveals lower sensitivity and a slightly poorer performance (more DEGs) of the alternative version. Table S3 contains the details for this alternative approach.
(PDF)

Table S1 **Data for each library in units of millions of reads.** The raw reads are split into quality reads and low quality/short reads. The quality reads are split into unmapped reads and mapped reads. The mapped reads are split into ribosomal reads and non-ribosomal reads. Finally the non-ribosomal reads are split into duplicate reads and useable reads. The low quality/short reads, unmapped reads, ribosomal reads and the duplicate reads are all discarded from analysis.
(XLSX)

Table S2 **List of non-histone protein coding genes without poly A tail.** From the differential expression between the groups RIN 10 and RiboMinus (RIN 2-6) we identify genes that have much higher expression in the RiboMinus group than in the RIN 10 group. Many of those genes are histone genes, known to be without poly A tail. This table lists other genes that potentially also lack a poly A tail judging from their expression profile (Figure 6c).
(XLSX)

Table S3 **Effects of the 3TC method on differential expression for various length restrictions.** Here the isoform filtering is done by selecting the isoform which has the highest expression in the RIN 10 group.
(XLSX)

Table S4 **Effects of the 3TC method on differential expression for various length restriction using alternative isoform filtering approach.** Here the isoform filtering is done by selecting the isoform/s which is/are closest to the 3' end of the gene.
(XLSX)
