# Supplementary material for: Sequencing Degraded RNA Addressed by 3' Tag Counting
Source: PLoS One. 2014 Mar 14;9(3):e91851. doi: 10.1371/journal.pone.0091851 (PMC3954844; doi:10.1371/journal.pone.0091851)
Supplement: Figure S1 — Flowchart of the two library preparations protocols used. a) poly-A enrichment and b) ribosomal depletion. The steps shaded with blue have been automated on a Magnatrix 1200 Biomagnetic Workstation. After RNA extraction the RNA is run through a quality check (QC) with a BioAnalyzer and Qubit quantification fluorometer. The dotted QC line indicates that it is only safe to continue with the procedure depending on the results from the quality check. The cDNA libraries go through the same quality check procedure after the CA purification (The CA purification is a washing step based on carboxil acid beads). The alterations from the standard protocol lie in the automation and the CA purification as well as the ribosomal depletion for the protocol in b). (PDF) [file pone.0091851.s001.pdf]

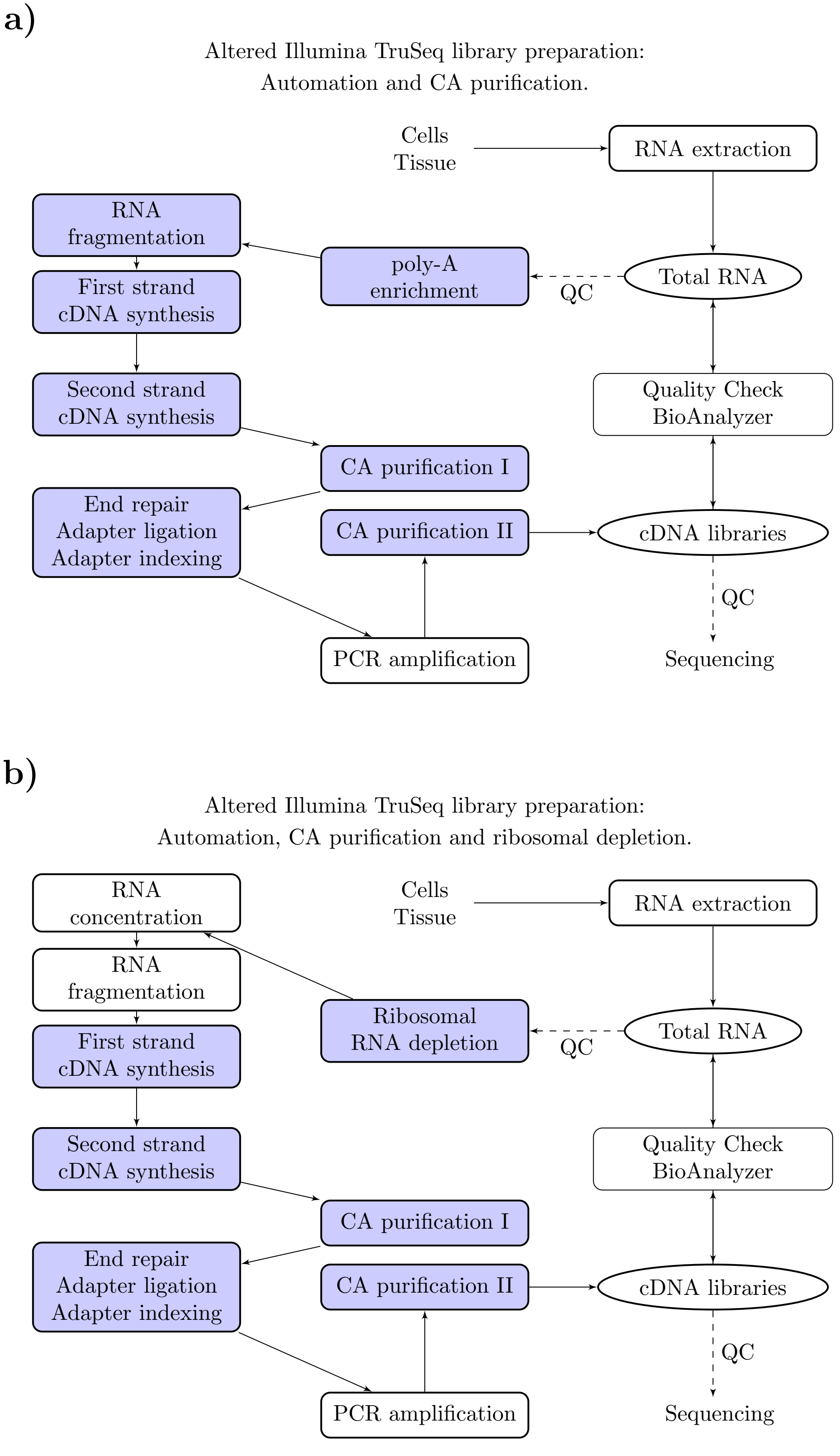
a)
Altered Illumina TruSeq library preparation:
Automation and CA purification.
Cells
Tissue
RNA extraction
RNA fragmentation
poly-A enrichment
Total RNA
QC
First strand cDNA synthesis
Second strand cDNA synthesis
Quality Check BioAnalyzer
CA purification I
End repair
Adapter ligation
Adapter indexing
CA purification II
cDNA libraries
QC
PCR amplification
Sequencing
b)
Altered Illumina TruSeq library preparation:
Automation, CA purification and ribosomal depletion.
RNA concentration
Cells
Tissue
RNA extraction
RNA fragmentation
Ribosomal RNA depletion
Total RNA
QC
First strand cDNA synthesis
Second strand cDNA synthesis
Quality Check BioAnalyzer
CA purification I
End repair
Adapter ligation
Adapter indexing
CA purification II
cDNA libraries
QC
PCR amplification
Sequencing
