# Supplementary figures and images for: Sequencing Degraded RNA Addressed by 3' Tag Counting
Source: PLoS One. 2014 Mar 14;9(3):e91851. doi: 10.1371/journal.pone.0091851 (PMC3954844; doi:10.1371/journal.pone.0091851)

## Original GTF

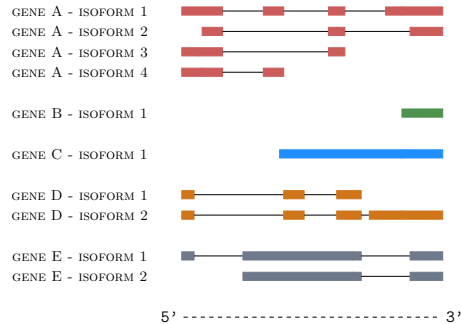

isoform filtering

## 3TC process

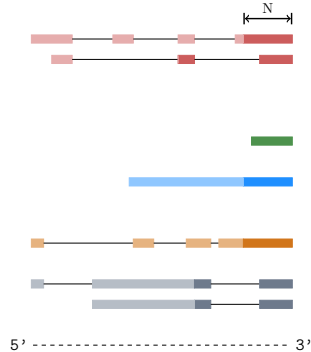

length restriction

## Final GTF

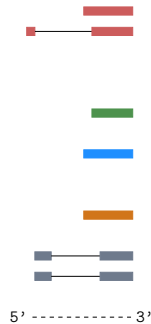

Supplement: Figure S2 — Schematic diagram of the alternative isoform filtering for 3TC. Alternative isoform filtering for 3TC, independent of samples used. Instead of determining the highest expressed isoform, within a gene, from the group with the highest quality, we select the isoforms that are closest to the three prime end. This approach is simpler in the sense that here it is not necessary to determine the highest expressed isoform within a gene and it is independent of the expression of the samples used. However it does not perform as well since it will, occasionally, select isoforms that are lowly or not expressed. For results using this isform filtering approach, see Figure S8. (PDF) [file pone.0091851.s002.pdf]

# Effects of preprocessing analysis pipeline

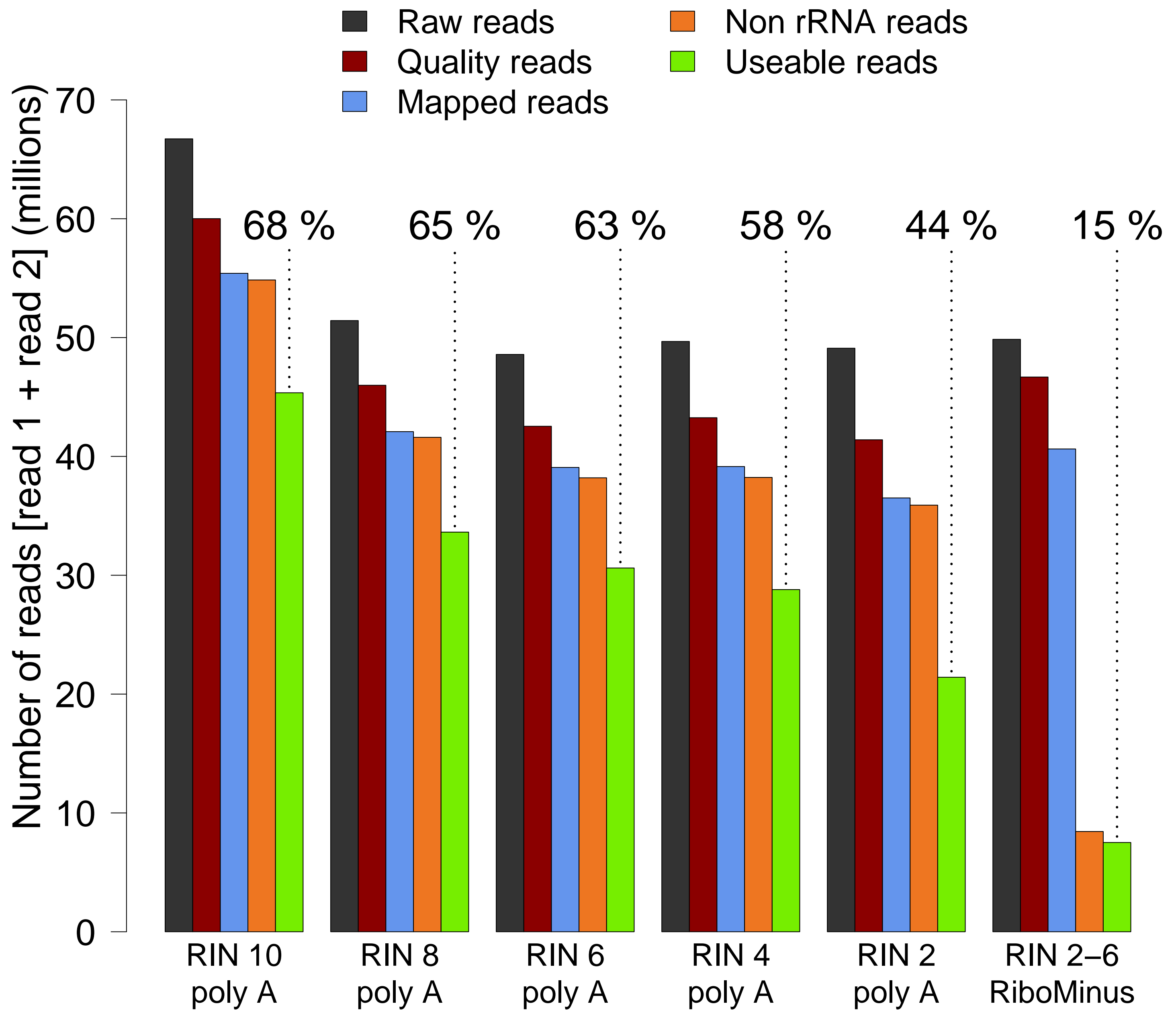

Supplement: Figure S3 — Preprocessing of sequencing data with duplicate removal. The barplot shows how many reads survive through each of the steps of the preprocessing pipline, as Figure 4 in the main text, with an additonal step of removing duplicates - from Non rRNA read to Useable reads. A large amount of reads are lost due to rRNA read removal in the RiboMinus group. The percentage of useable reads (shown above the dotted lines) shows a steady decline with decreasing RIN. This is a cumulative effect of each step but is mostly due to increasing amount of duplicates with lower RIN. A one-way ANOVA finds a statistically significant difference in useable reads between the RIN groups (F = 64.4, p0.00001) and a Tukey HSD test reveals a statisitical difference between all groups (p0.05) except between the RIN 10 and RIN 8 groups and between the RIN 8 and RIN 6 groups. The poor performance of the RiboMinus samples can be attributed to high rRNA contamination. (PDF) [file pone.0091851.s003.pdf]

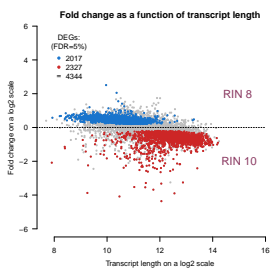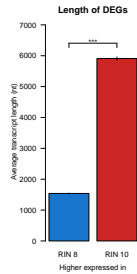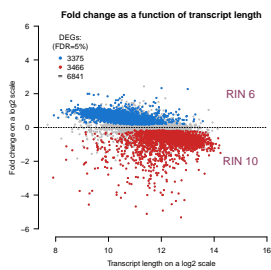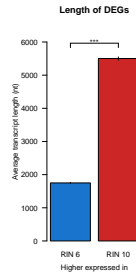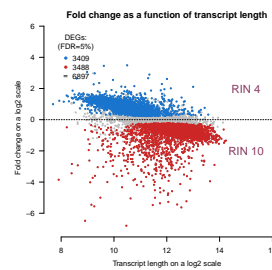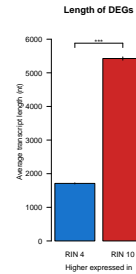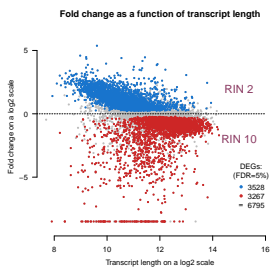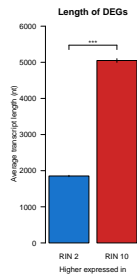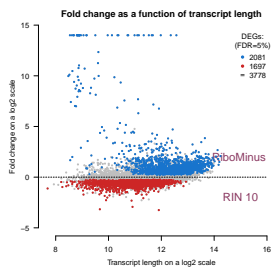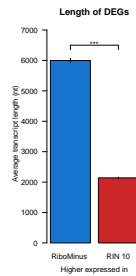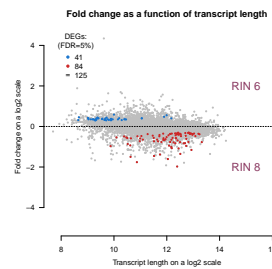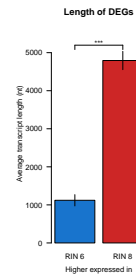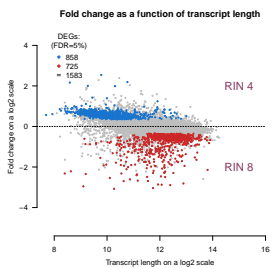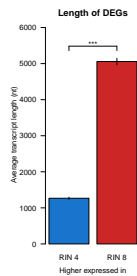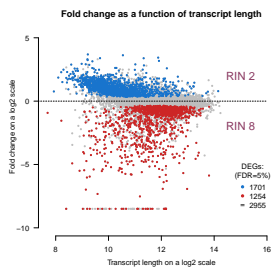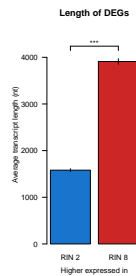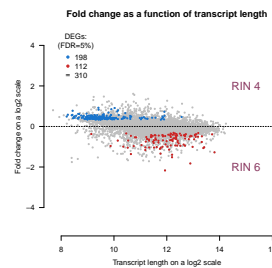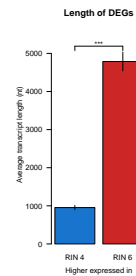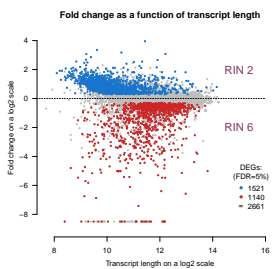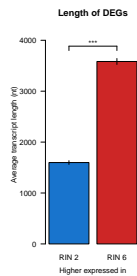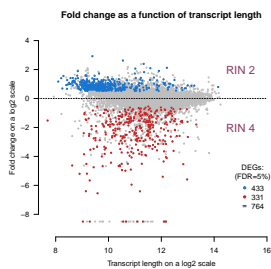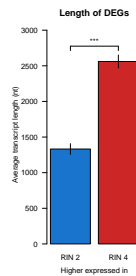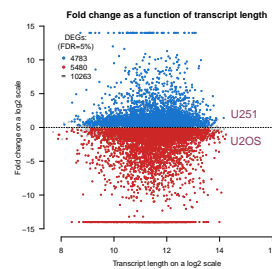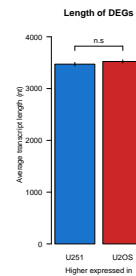

Supplement: Figure S5 — Differential expression profiles between the experimental groups. In almost all instances the DEGs that are more highly expressed in the group with higher RIN are, on average, significantly longer than the DEGs that are more highly expressed in the group with lower RIN. The two exceptions are the comparison between RIN 10 and RiboMinus where it is the opposite and the control group where there is no difference in the length of DEGs. (PDF) [file pone.0091851.s005.pdf]

# Density distribution of gene lengths for differentially expressed genes (RIN 10 vs. RIN 8)

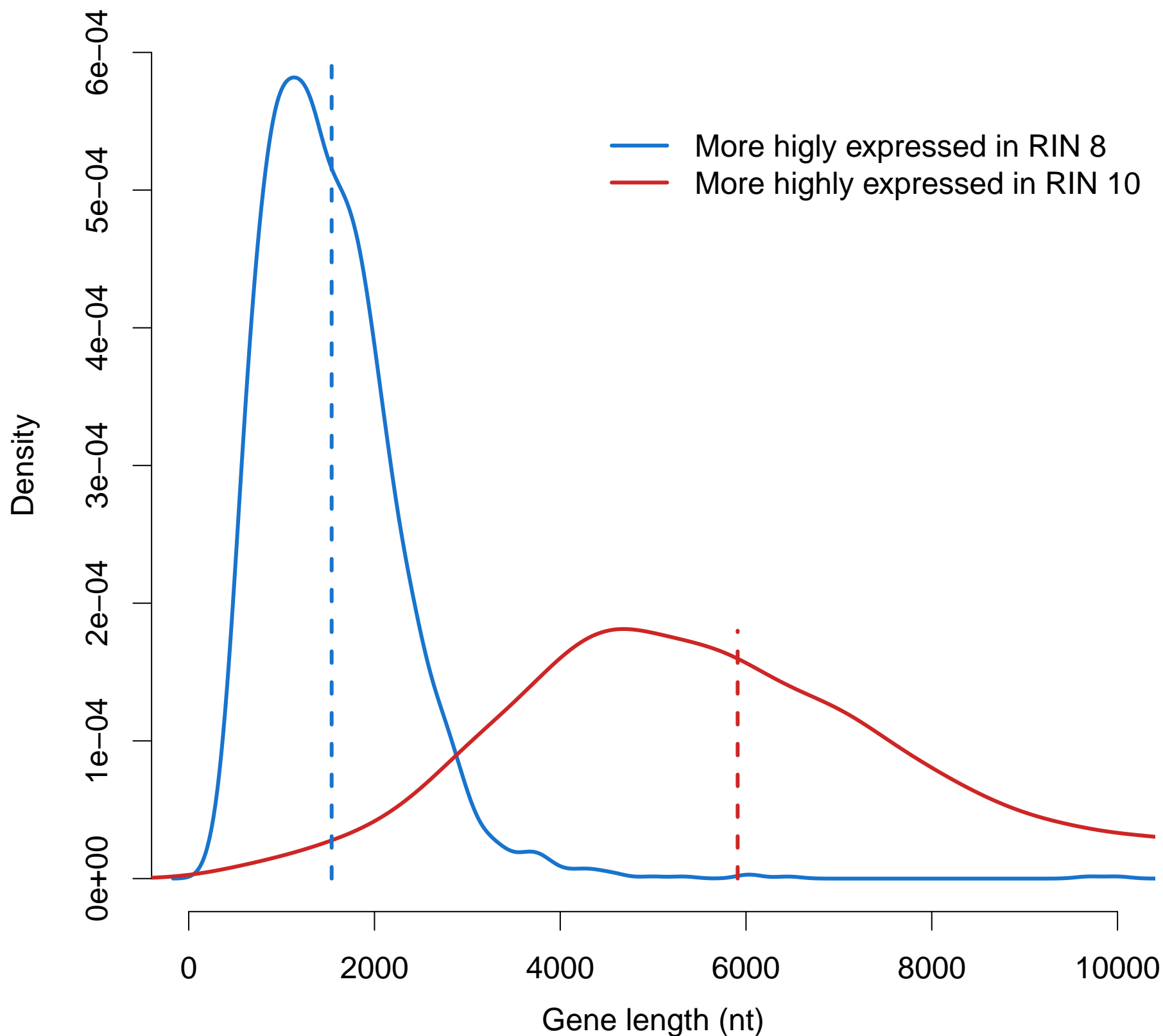

Supplement: Figure S6 — The length distribution of up and down regulated DEGs from the comparison RIN 10 vs. RIN 8. The dotted vertical lines show the means depicted in Figure 6b. (PDF) [file pone.0091851.s006.pdf]

# rRNA reads in samples with low and high RIN after being treated with RiboMinus

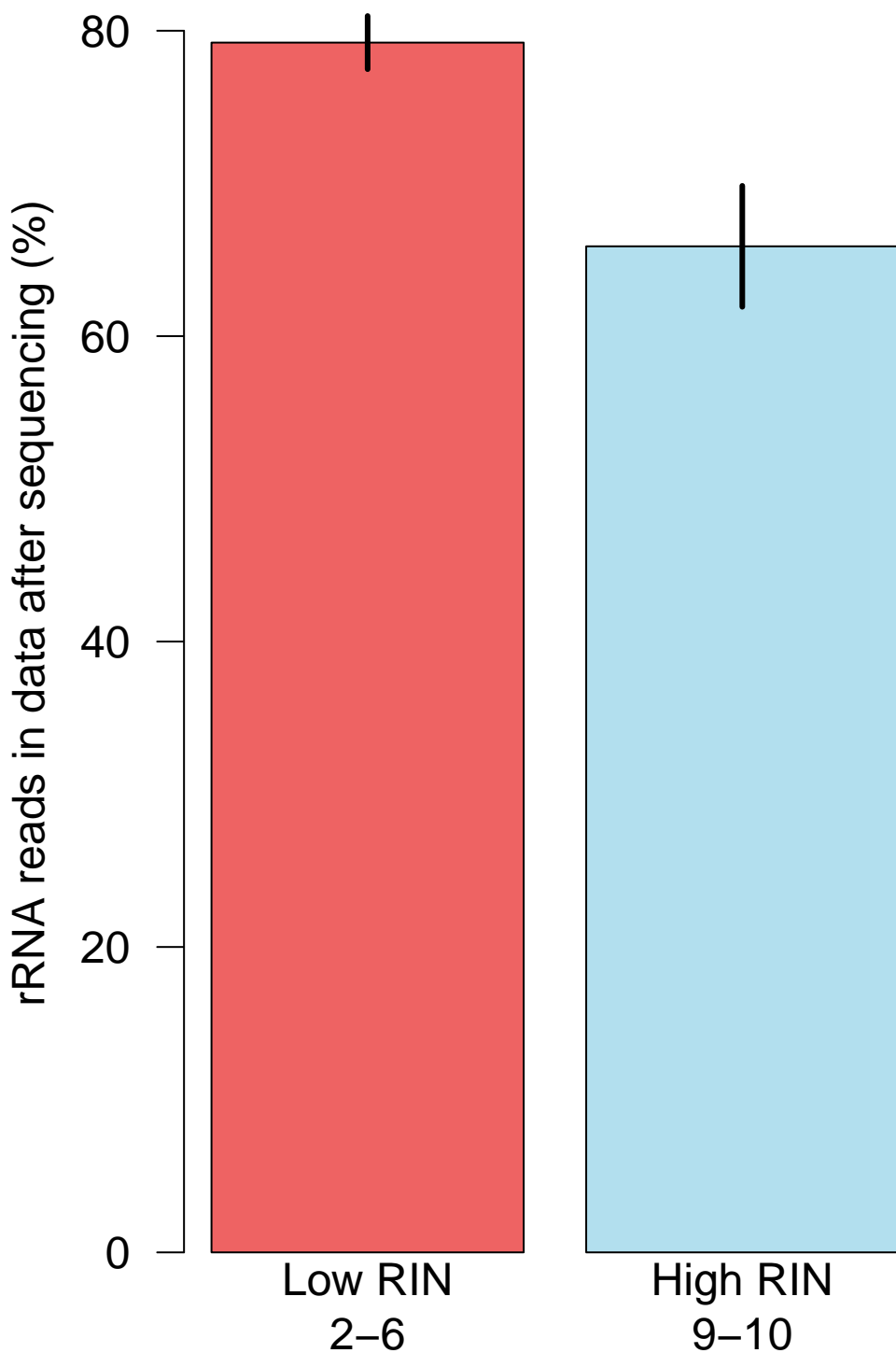

Supplement: Figure S7 — Effects of RiboMinus of low and high quality samples. Ribosomal RNA in samples with low and high RIN values after being treated with the RiboMinus kit. The samples in the Low RIN bar are the same RiboMinus samples used in the current study. The sampels representing the High RIN are from another unpuplished study using the same ribosomal depletion method. The Low RIN bar is the average from four samples while the High RIN bar is an average from six samples. The error bars show the standard error. The RiboMinus kit performs signficantly better on high quality samples (p0.05, Student's t-test) but the High RIN samples still contain above 65% of rRNA reads which is must be considered unacceptable. (PDF) [file pone.0091851.s007.pdf]
