# Supplementary material for: Sequencing Degraded RNA Addressed by 3' Tag Counting
Source: PLoS One. 2014 Mar 14;9(3):e91851. doi: 10.1371/journal.pone.0091851 (PMC3954844; doi:10.1371/journal.pone.0091851)
Supplement: Figure S4 — Venn diagrams showing the effects of isoform filetering on differential expression. The number and overlap of DEGs between two methods counting for differential expression. One method 'No 3TC' (light green) counts towards an unaltered gtf annotation file as downloaded from ensembl.org. The other method '3TC - isoform filtering' (light red) uses an annotation file that has gone through the isoform filtering step of the 3TC method but no length restriction. In general there is good agreement with the two methods but the '3TC - isoform filtering' usually has more unique DEGs than the 'No 3TC' method. (These Venn diagrams are effectively visualizations of Table 1 from the main text.). (PDF) [file pone.0091851.s004.pdf]

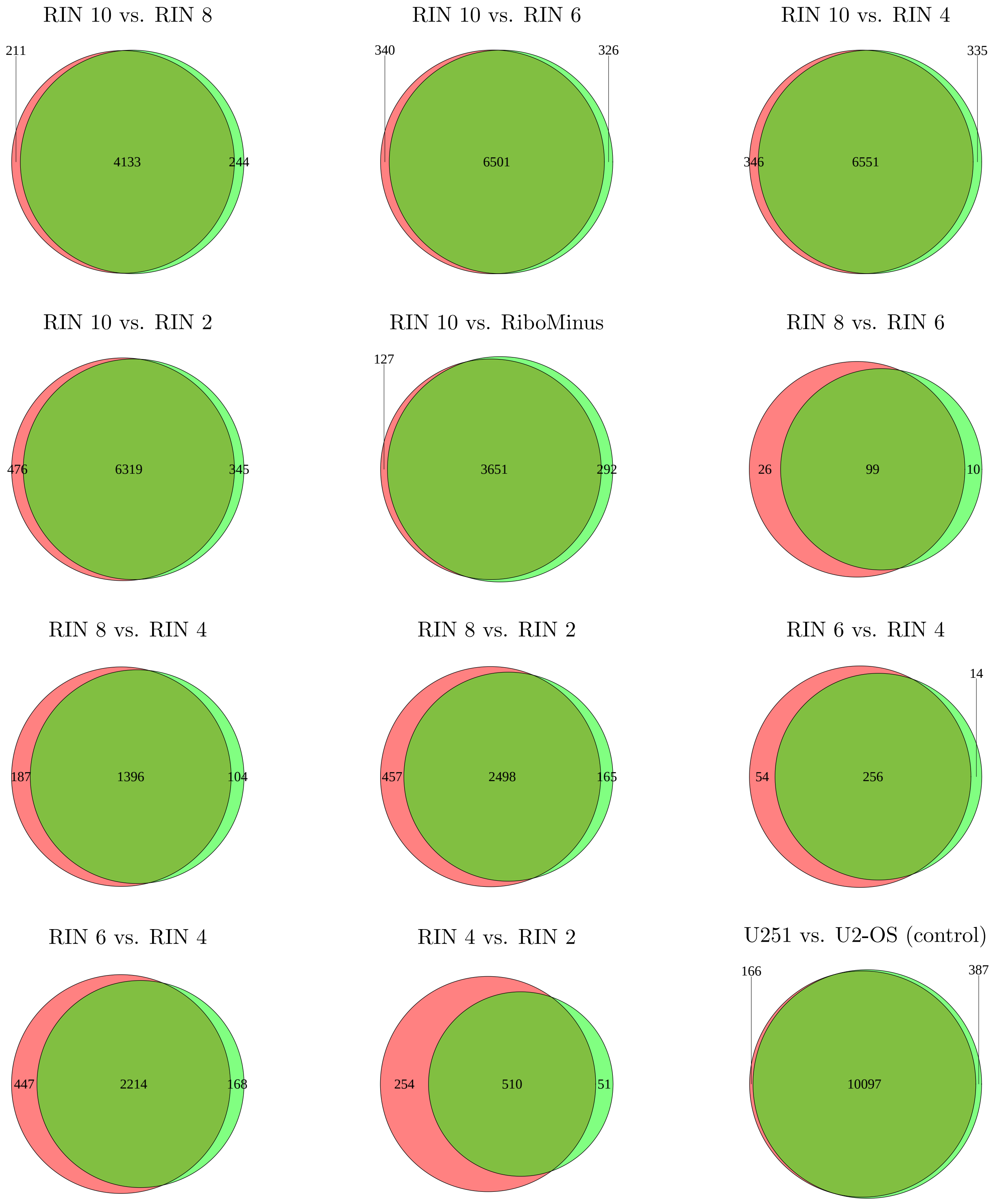
RIN 10 vs. RIN 8
211
4133
244
RIN 10 vs. RIN 6
340
326
6501
RIN 10 vs. RIN 4
335
346
6551
RIN 10 vs. RIN 2
476
6319
345
RIN 10 vs. RiboMinus
127
3651
292
RIN 8 vs. RIN 6
26
99
10
RIN 8 vs. RIN 4
187
1396
104
RIN 8 vs. RIN 2
457
2498
165
RIN 6 vs. RIN 4
14
54
256
RIN 6 vs. RIN 4
447
2214
168
RIN 4 vs. RIN 2
254
510
51
U251 vs. U2-OS (control)
166
387
10097
