# Supplementary material for: Sequencing Degraded RNA Addressed by 3' Tag Counting
Source: PLoS One. 2014 Mar 14;9(3):e91851. doi: 10.1371/journal.pone.0091851 (PMC3954844; doi:10.1371/journal.pone.0091851)
Supplement: Figure S8 — Comparison between two versions of the isoform filtering step of the 3TC method. a) and b) is a reproduction of Figure 8 from the main text. c) and d) show the results using the alternative isoform selection explained in Figure S2. The versions show similar results but scrutinization reveals lower sensitivity and a slightly poorer performance (more DEGs) of the alternative version. Table S3 contains the details for this alternative approach. (PDF) [file pone.0091851.s008.pdf]

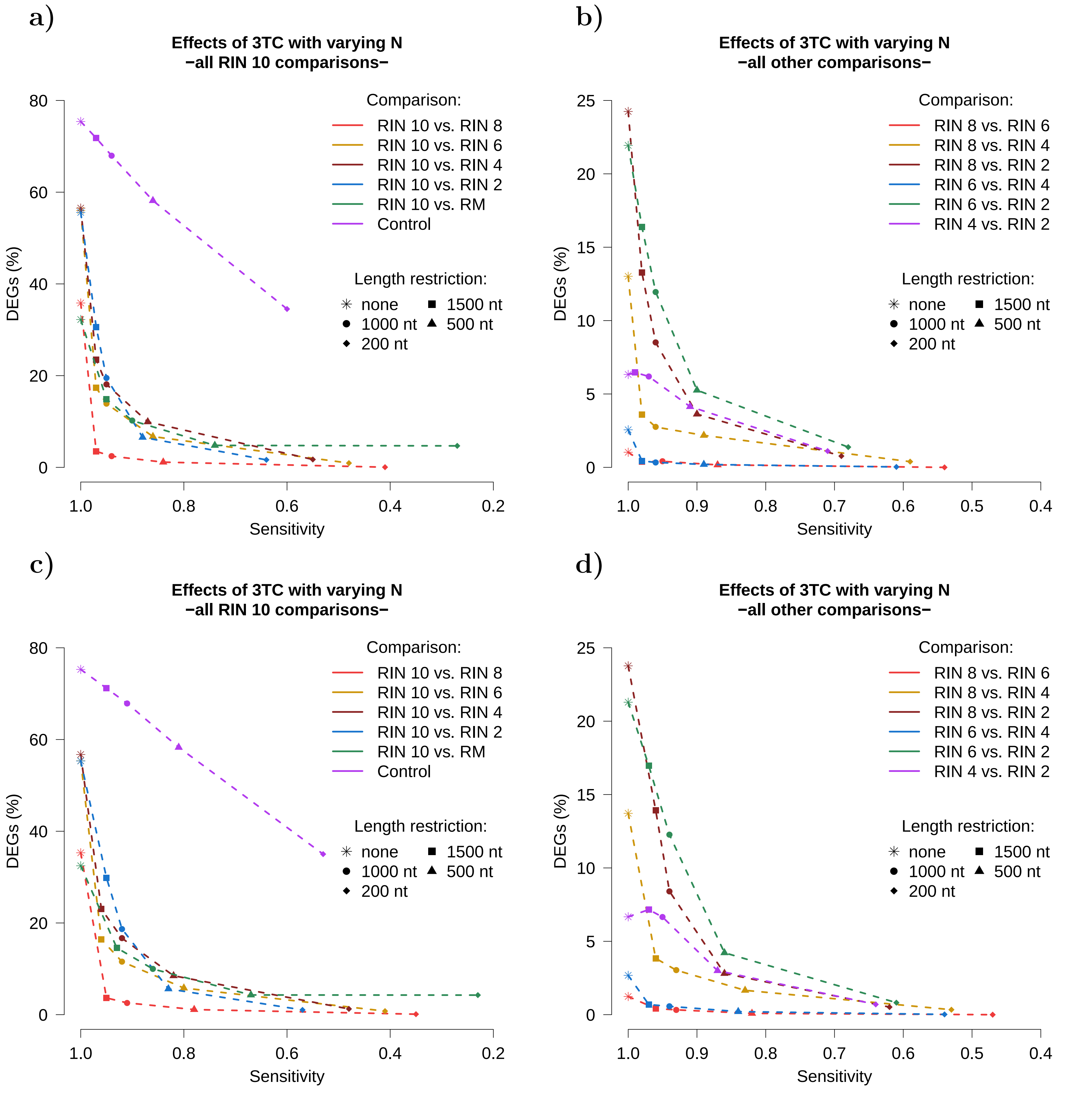

a)
Effects of 3TC with varying N
−all RIN 10 comparisons−
Comparison:
RIN 10 vs. RIN 8
RIN 10 vs. RIN 6
RIN 10 vs. RIN 4
RIN 10 vs. RIN 2
RIN 10 vs. RM
Control
Length restriction:
none
1500 nt
1000 nt
500 nt
200 nt
DEGs (%)
Sensitivity
b)
Effects of 3TC with varying N
−all other comparisons−
Comparison:
RIN 8 vs. RIN 6
RIN 8 vs. RIN 4
RIN 8 vs. RIN 2
RIN 6 vs. RIN 4
RIN 6 vs. RIN 2
RIN 4 vs. RIN 2
Length restriction:
none
1500 nt
1000 nt
500 nt
200 nt
DEGs (%)
Sensitivity
c)
Effects of 3TC with varying N
−all RIN 10 comparisons−
Comparison:
RIN 10 vs. RIN 8
RIN 10 vs. RIN 6
RIN 10 vs. RIN 4
RIN 10 vs. RIN 2
RIN 10 vs. RM
Control
Length restriction:
none
1500 nt
1000 nt
500 nt
200 nt
DEGs (%)
Sensitivity
d)
Effects of 3TC with varying N
−all other comparisons−
Comparison:
RIN 8 vs. RIN 6
RIN 8 vs. RIN 4
RIN 8 vs. RIN 2
RIN 6 vs. RIN 4
RIN 6 vs. RIN 2
RIN 4 vs. RIN 2
Length restriction:
none
1500 nt
1000 nt
500 nt
200 nt
DEGs (%)
Sensitivity
